# Depressive Symptom Burden and Its Relationship With Health-Related Quality of Life in Patients on Peritoneal Dialysis

**DOI:** 10.7759/cureus.106297

**Published:** 2026-04-01

**Authors:** Cecilia Salud-Gutiérrez, Ulises Avedaño-Ignacio, Merhary B Meza-Matus, Alfredo González-Ayala, Nallely Rincón Peregrino, Quitzia L Torres-Salazar

**Affiliations:** 1 Family Medicine, Hospital General de Zona No. 2 con Medicina Familiar, Instituto Mexicano del Seguro Social, Salina Cruz, MEX; 2 Biomedical Sciences, Universidad Juárez del Estado de Durango, Durango, MEX

**Keywords:** chronic kidney disease, depression, depressive symptoms, health-related quality of life, kdqol-36, peritoneal dialysis, phq-9, quality of life

## Abstract

Background

Chronic kidney disease (CKD) requiring peritoneal dialysis imposes a substantial physical, emotional, and social burden on patients. Among the non-biological factors influencing patient outcomes, depressive symptoms have emerged as a key determinant of health-related quality of life (HRQoL). However, the relationship between depression and HRQoL has not been fully characterized in patients undergoing peritoneal dialysis, particularly in Latin American populations.

Objective

To evaluate how depressive symptoms relate to HRQoL in patients with CKD undergoing peritoneal dialysis.

Methods

An observational, cross-sectional study was conducted at a second-level healthcare center of the Mexican Social Security Institute (IMSS) between June and December 2025. A total of 150 adult patients undergoing peritoneal dialysis were included. HRQoL was assessed using the Kidney Disease Quality of Life-36 (KDQOL-36) instrument, and depressive symptoms were evaluated using the Patient Health Questionnaire-9 (PHQ-9). Descriptive statistics were expressed as medians and interquartile ranges (IQR; q25-q75) or frequencies and percentages. Spearman’s rank correlation coefficient was used to evaluate the relationship between variables.

Results

The median HRQoL score was 53.19 (q25-q75: 38.7-65.7), and the median PHQ-9 score was seven (q25-q75: 4-11), corresponding predominantly to mild depressive symptoms. A strong and statistically significant inverse relationship was observed between depressive symptoms and HRQoL (rₛ = −0.771, p<0.001). Additionally, a progressive decline in quality of life scores was identified with increasing severity of depressive symptoms, demonstrating a clear gradient effect.

Conclusion

Depressive symptoms are strongly related to impaired HRQoL among patients receiving peritoneal dialysis. These findings highlight the importance of incorporating routine mental health assessment and multidisciplinary interventions into nephrology care to improve patient-centered outcomes.

## Introduction

Chronic kidney disease (CKD) remains a significant challenge for global public health, characterized by progressive and irreversible loss of renal function and a high prevalence of comorbid conditions that significantly impact patient outcomes. In advanced stages, the need for renal replacement therapy introduces additional physiological and psychosocial stressors, particularly in patients undergoing peritoneal dialysis [1]. While this modality offers greater autonomy and flexibility compared to hemodialysis, it also imposes substantial demands on daily life, including strict treatment adherence, lifestyle modifications, and continuous self-management [2]. Consequently, the clinical approach to CKD has evolved from a purely biomedical model toward a more comprehensive framework that incorporates patient-reported outcomes, especially health-related quality of life (HRQoL), as a key indicator of disease burden and treatment effectiveness [3].

Depression is one of the most prevalent yet frequently underrecognized comorbidities among patients with CKD undergoing dialysis, with prevalence rates markedly exceeding those observed in the general population. Importantly, in this context, depression should not be regarded merely as a psychological response to chronic illness, but rather as a multifactorial condition driven by a complex interplay of biological, inflammatory, neuroendocrine, and psychosocial mechanisms intrinsic to CKD [4]. The overlap between uremic symptoms and depressive manifestations further complicates its identification, often leading to underdiagnosis and undertreatment. Emerging evidence suggests that depressive symptoms are associated with poorer adherence to treatment, increased healthcare utilization, and adverse clinical outcomes, highlighting their relevance as a modifiable determinant within the continuum of CKD care [5].

Despite the growing recognition of the interplay between mental health and clinical outcomes in CKD, there remains a relative scarcity of data specifically addressing patients undergoing peritoneal dialysis, particularly in Latin American populations [6]. Moreover, this relationship has not been fully characterized in this subgroup, limiting the development of targeted, patient-centered interventions. Therefore, this study aimed to assess the relationship between depressive symptoms and health-related quality of life among patients receiving peritoneal dialysis in a tertiary care setting.

## Materials and methods

Study design and settings

The study adhered to Strengthening the Reporting of Observational Studies in Epidemiology (STROBE) reporting guidelines and the recommendations of the Enhancing the QUAlity and Transparency Of health Research (EQUATOR) Network for observational prevalence research [7]. An observational, cross-sectional study was conducted to evaluate the relationship between depressive symptoms and health-related quality of life in patients with CKD undergoing peritoneal dialysis. The study was carried out in a second-level healthcare center of the Mexican Social Security Institute (IMSS). Data collection was performed between June and December 2025.

Participants

The study population consisted of adult patients diagnosed with CKD undergoing peritoneal dialysis (continuous ambulatory or automated modalities) who attended follow-up consultations during the study period. Inclusion criteria comprised patients aged ≥18 years with established peritoneal dialysis treatment and the ability to complete the assessment instruments. Patients with cognitive impairment, previously diagnosed severe psychiatric disorders, or incomplete data were excluded. The sample size was calculated using the formula for proportions in finite populations, assuming a 95% confidence level. An expected prevalence of clinically relevant depressive symptoms of 32%, based on prior evidence in patients undergoing renal replacement therapy, was used. With a precision of 5%, the minimum required sample size was estimated at 143 participants.

Participants were recruited using a non-probabilistic consecutive sampling approach; all eligible patients attending follow-up consultations during the study period were consecutively invited to participate until the required sample size was reached. A total of 150 patients were included in the final analysis.

Variables and data collection

Sociodemographic and clinical variables were collected, including age, sex, dialysis modality, time on dialysis, and dialysis-related complications. HRQoL was assessed using the Kidney Disease Quality of Life-36 (KDQOL-36), a validated, self-administered instrument specifically designed for patients with CKD. The KDQOL-36 includes 36 items that evaluate both general health status and kidney disease-specific domains, including symptoms/problems, effects of kidney disease, burden of kidney disease, and physical and mental component summaries. Higher scores indicate better perceived quality of life [8,9]. Depressive symptoms were measured using the Patient Health Questionnaire-9 (PHQ-9), a validated self-administered instrument consisting of nine items that assess the frequency of depressive symptoms over the previous two weeks. Scores range from 0 to 27, with higher scores indicating greater severity of depressive symptoms [10]. Depression severity was categorized according to standard PHQ-9 cut-off points (minimal, mild, moderate, moderately severe, and severe).

Statistical analysis

Descriptive statistics were used to summarize the data. Continuous variables were expressed as median and interquartile range (q25-q75), while categorical variables were presented as frequencies and percentages. The relationship between depressive symptoms and quality of life scores was evaluated using Spearman’s rank correlation coefficient (rₛ), given the non-normal distribution of the variables. A p-value <0.05 was considered statistically significant. Statistical analyses were performed using IBM SPSS Statistics for Windows, Version 27 (Released 2020; IBM Corp., Armonk, New York, United States; Spanish version).

Ethical considerations

The study received approval from the Research and Ethics Committee of the IMSS (protocol number R-2024-2001-050). All procedures were performed in accordance with the principles of the Declaration of Helsinki. Written informed consent was obtained from all participants prior to their inclusion.

## Results

A total of 150 patients undergoing peritoneal dialysis were included in the analysis. The median age was 54 years (q25-q75: 44-63), with an equal distribution by sex, with 75 male subjects (50%) and 75 female subjects (50%). Most patients were receiving continuous ambulatory peritoneal dialysis (CAPD/DPCA) (n=116, 77.3%), while 34 (22.7%) were on automated peritoneal dialysis (APD/DPA). Regarding dialysis duration, the largest proportions of patients had been on therapy for one year (n=49, 32.7%) or ≥three years (n=49, 32.7%). More than half of the patients (n=85; 56.7%) reported no dialysis-related complications, whereas catheter dysfunction was observed in 28 (18.7%), peritonitis in 12 (8.0%), and other complications in 25 (16.7%) patients (Table 1).

**Table 1:** Demographic, clinical characteristics, and patient-reported outcomes of patients undergoing peritoneal dialysis (n=150) Continuous variables are presented as median (q25–q75), and categorical variables as n (%). Quality of life was assessed using the Kidney Disease Quality of Life-36 (KDQOL-36) instrument, and depressive symptoms were evaluated using the Patient Health Questionnaire-9 (PHQ-9).

Variable	Result
Age (years), median (q25–q75)	54 (44-63)
Sex, n (%)	
Male	75 (50%)
Female	75 (50%)
Peritoneal dialysis modality, n (%)	
Continuous ambulatory peritoneal dialysis (CAPD/DPCA)	116 (77.3%)
Automated peritoneal dialysis (APD/DPA)	34 (22.7%)
Time on peritoneal dialysis, n (%)	
<1 year	26 (17.3%)
1 year	49 (32.7%)
2 years	26 (17.3%)
≥3 years	49 (32.7%)
Dialysis-related complications, n (%)	
No complications	85 (56.7%)
Catheter dysfunction	28 (18.7%)
Peritonitis	12 (8.0%)
Other complications	25 (16.7%)
Patient-reported outcomes, median (q25–q75)	
Quality of life (KDQOL-36)	53.19 (38.7–65.7)
Depression score (PHQ-9)	7 (4–11)

The overall median HRQoL score measured by KDQOL-36 was 53.19 (q25-q75: 38.7-65.7), indicating a moderate level of perceived well-being. The median PHQ-9 score was seven (q25-q75: 4-11), corresponding predominantly to mild depressive symptomatology. When stratified by severity, minimal depression was observed in 50 patients (33.3%) and mild depression in 47 (31.3%), representing the majority of the sample, while moderate to severe forms were less common (Table 2).

**Table 2:** Severity of depressive symptoms and domain-specific health-related quality of life in patients undergoing peritoneal dialysis Depression severity was classified according to the Patient Health Questionnaire-9 (PHQ-9) score. Quality of life domains were assessed using the Kidney Disease Quality of Life-36 (KDQOL-36) instrument. Continuous variables are presented as median (q25–q75), and categorical variables as percentages.

Depression severity, n (%)	
Minimal	50 (33.3%)
Mild	47 (31.3%)
Moderate	33 (22.0%)
Moderately severe	16 (10.7%)
Severe	4 (2.7%)
KDQOL-36 domain scores, median (q25–q75)	
Symptoms	79.2 (62.5–89.6)
Mental component	65.0 (48.3–75.8)
Physical component	43.3 (30.6–59.4)
Effects of disease	46.9 (31.3–68.8)
Burden of disease	25.0 (6.3–50.0)

Analysis of KDQOL-36 domains showed heterogeneity across dimensions. Higher scores were observed in the symptoms domain (79.2 (62.5-89.6)) and mental component (65.0 (48.3-75.8)), whereas lower scores were identified in the physical component (43.3 (30.6-59.4)), effects of disease (46.9 (31.3-68.8)), and particularly in the burden of disease domain (25.0 (6.3-50.0)), suggesting a substantial perceived impact of CKD on daily life (Table 2).

**Figure 1:**
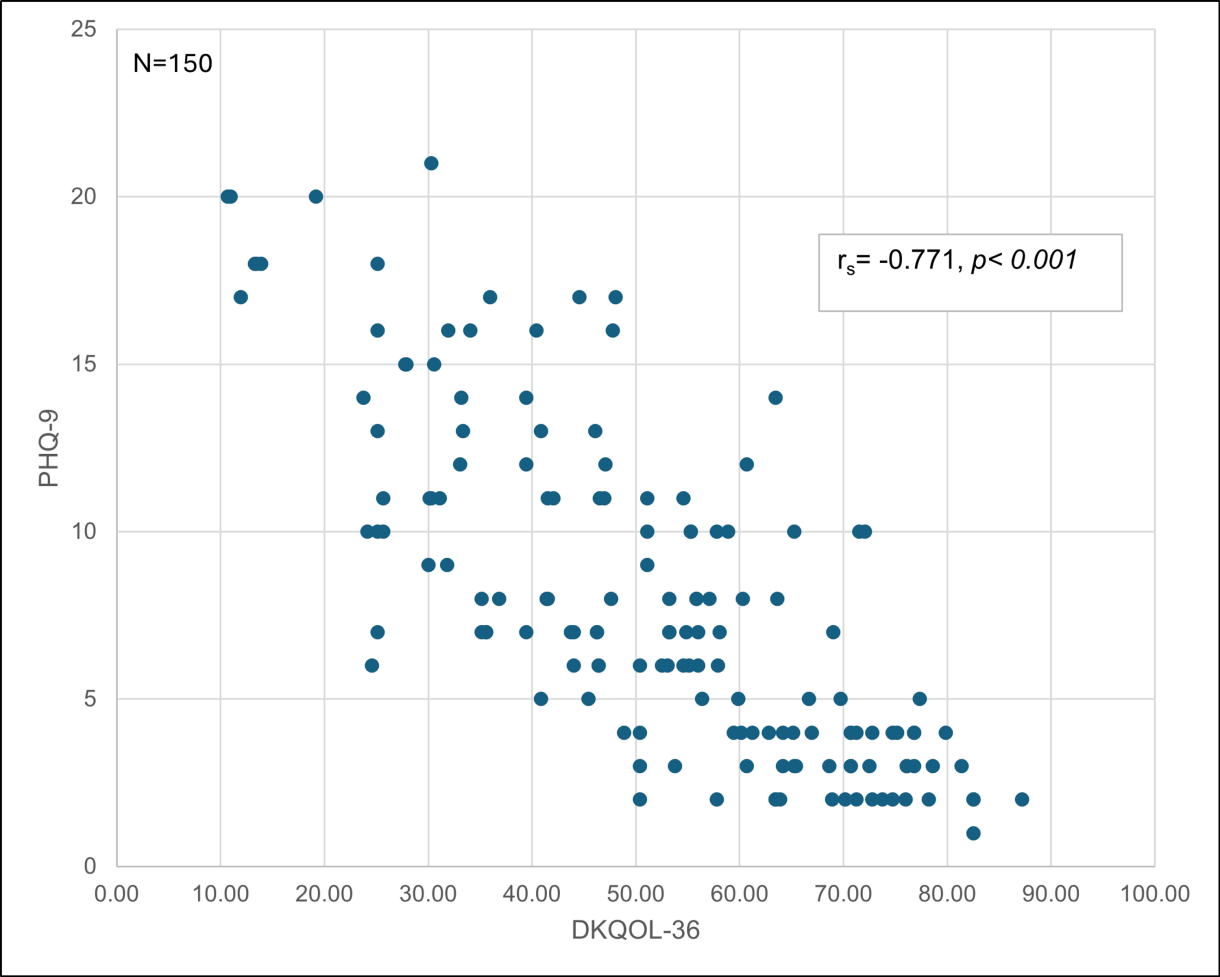
Correlation between health-related quality of life (KDQOL-36) and depressive symptoms (PHQ-9) in patients undergoing peritoneal dialysis Spearman correlation analysis demonstrated a strong and statistically significant inverse relationship between  Kidney Disease Quality of Life-36 (KDQOL-36) scores and Patient Health Questionnaire-9 (PHQ-9) scores (rₛ = −0.771, p<0.001). Higher depressive symptom severity was associated with lower health-related quality of life. Each dot represents an individual patient (n=150).

A Spearman correlation analysis demonstrated a strong and statistically significant inverse relationship between KDQOL-36 scores and PHQ-9 scores (rₛ = −0.771, p<0.001). As depressive symptom severity increased, quality of life consistently decreased. The dispersion pattern confirmed a clear downward trend, supporting the role of depression as a key factor associated with reduced physical, mental, and social well-being in this population (Figure 1).

## Discussion

The present study demonstrates a strong and statistically significant inverse association between depressive symptoms and HRQoL among patients with CKD receiving peritoneal dialysis (rₛ = −0.771; p<0.001). This finding not only confirms the initial hypothesis but also provides robust quantitative evidence supporting depression as a central determinant of reduced well-being in this population. The magnitude of the observed correlation suggests that depression should not be considered a secondary or reactive condition, but rather a key factor influencing functional, emotional, and social domains in patients with advanced kidney disease.

These results are consistent with previous international studies that have identified depression as one of the strongest predictors of impaired health-related quality of life in patients receiving renal replacement therapy. Perales-Montilla et al. reported that depressive symptoms were independently associated with lower KDQOL-36 scores, even after adjustment for clinical and sociodemographic variables [11]. Similarly, Mohamed et al. found significant negative correlations between PHQ-9 scores and multiple domains of quality of life, with coefficients ranging from −0.40 to −0.69 [12]. Compared to these reports, the strength of the relationship observed in our study appears even greater, reinforcing the clinical relevance of depressive symptomatology as a major contributor to perceived health deterioration across different healthcare settings.

The overall quality of life in our cohort was moderate, with a median KDQOL-36 score of 53.19, and showed a heterogeneous distribution across domains. Higher scores were observed in the symptoms and mental domains, whereas lower scores were found in the physical component, effects of disease, and particularly in the burden of disease domain. This pattern reflects the substantial impact of CKD and its treatment on daily functioning and aligns with previous findings in Latin American and African populations, where disease burden and treatment-related limitations have been consistently identified as the most affected dimensions of quality of life [6,12].

Importantly, the stratified analysis revealed a clear gradient in quality of life according to depression severity, demonstrating a dose-response relationship. Patients with minimal depressive symptoms exhibited substantially higher quality of life scores, whereas those with severe depression showed a marked deterioration. This progressive decline supports the notion that even mild depressive symptoms may have clinically meaningful effects, which intensify as symptom severity increases. Similar patterns have been described by Mohamed et al. and Mathew et al., further supporting the consistency of this relationship across different populations and dialysis modalities [12,13].

From a pathophysiological perspective, the relationship between depression and reduced quality of life in CKD is likely mediated by a complex interplay of biological and psychosocial mechanisms. Chronic inflammation, activation of the hypothalamic-pituitary-adrenal axis, and alterations in serotonergic and dopaminergic pathways have all been implicated in the development of depressive symptoms in this population. These processes contribute to fatigue, anhedonia, sleep disturbances, and reduced functional capacity, which directly impact patients’ perception of well-being. In addition, the psychosocial burden of dialysis, including treatment adherence, lifestyle restrictions, and reduced autonomy, further amplifies emotional distress and social isolation [14].

The role of contextual and socioeconomic factors should also be considered. While studies conducted in low-resource settings have highlighted the impact of economic constraints on quality of life, the population in our study (benefiting from institutional healthcare coverage) still exhibited significant levels of depression. This suggests that, even in settings with improved access to care, psychological factors remain a critical and often under-addressed component of disease burden.

These findings have important clinical implications. Routine screening for depressive symptoms using validated instruments such as the PHQ-9, along with the assessment of quality of life through tools like KDQOL-36, should be integrated into standard care for patients undergoing peritoneal dialysis. Early identification of psychological distress may allow timely interventions, including multidisciplinary approaches involving mental health support, patient education, and social assistance, which may ultimately improve both quality of life and clinical outcomes [15].

This study has limitations. The cross-sectional design prevents causal interpretation between depressive symptoms and quality of life. Additionally, the results derive from a single-center population, which may limit their generalizability to other settings or healthcare systems. Furthermore, a potential for selection bias related to the patient inclusion process should be considered. Nevertheless, the relatively large sample size, the use of validated and widely recognized instruments, and the consistency of the findings with previously published literature provide robustness and credibility to the results.

Overall, our findings demonstrate a strong and clinically meaningful relationship between depressive symptoms and reduced health-related quality of life in patients with CKD undergoing peritoneal dialysis. The consistency and magnitude of this relationship highlight the importance of recognizing depression not merely as a comorbidity, but as a central component influencing patient well-being. These results support the need for a comprehensive biopsychosocial approach in nephrology care, in which mental health assessment and intervention are integrated as essential elements of routine clinical practice rather than secondary considerations.

## Conclusions

HRQoL in patients with CKD undergoing peritoneal dialysis is significantly impaired and demonstrates a strong inverse relationship with depressive symptom burden. The magnitude and consistency of this association across multiple domains of the KDQOL-36 highlight HRQoL as a critical patient-centered outcome that is closely linked to psychological factors. These findings emphasize that depressive symptoms are not merely comorbid conditions but key contributors to the overall perception of well-being in this population.

From a clinical perspective, the routine assessment of HRQoL using validated instruments such as the KDQOL-36, together with systematic screening for depressive symptoms, should be integrated into standard nephrology care. Addressing psychological distress through multidisciplinary and patient-centered interventions may play a fundamental role in improving quality of life and optimizing overall outcomes. Further longitudinal research is needed to clarify the directionality of this relationship and evaluate the impact of targeted interventions.
